# Distinct Patterns of Liver Chemistry Changes in Pediatric Acute Hepatitis of Unknown Origin and COVID-19 Patients: A Systematic Review

**DOI:** 10.7759/cureus.58307

**Published:** 2024-04-15

**Authors:** Carly Van Wylick, Lauren Lewis, Daniel J Mulder

**Affiliations:** 1 Pediatrics, Queen's University, Kingston, CAN

**Keywords:** liver disease etiology, pediatric hepatology, emerging communicable diseases, liver failure, disease outbreak

## Abstract

In 2021 and 2022, there were noted to be clusters of pediatric acute hepatitis of unknown origin (AHUO) occurring across the globe. While there was not necessarily a global increase in cases, understanding the pattern of liver injury in AHUO is crucial to properly identify cases of this unexplained phenomenon, especially since it occurred simultaneously with a global resurgence of COVID-19. The objective of this study was to contrast the patterns in liver-relevant biochemical data from COVID-19 patients and AHUO. Studies reporting liver chemistries for cases of AHUO and COVID-19 were identified by a systematic review and search of the literature. For each case, alanine aminotransferase (ALT), aspartate aminotransferase (AST), total bilirubin, direct bilirubin, and international normalized ratio (INR) levels were extracted as available. These were normalized to multiples of the upper limit of normal by patient age. There were statistically significant greater elevations of ALT and AST in patients with AHUO than in those with COVID-19. Only a subset of patients with COVID-19 had an AST or ALT greater than the normal range. INR elevation could be substantial for both conditions but was also statistically higher in the AHUO group. Liver chemistry changes were not statistically correlated with age. The pattern of liver chemistry changes between AHUO and COVID-19 have some distinctions, which suggests that AHUO is not a phenomenon driven primarily by SARS-CoV-2 infection alone. Differentiating AHUO and COVID-19 would be challenging based on patterns of liver chemistry changes alone.

## Introduction and background

There have recently been unexplained clusters of pediatric hepatitis worldwide. Between October 2021 and July 2022, over 1000 cases of pediatric acute hepatitis of unknown origin (AHUO) had been reported in 35 countries, with 5% requiring a liver transplant, and 2% being fatal [1]. This represented increased local incidence in certain areas compared to previous reports [2]. However, others have reported that AHUO-related hospitalizations have not increased from the pre-pandemic baselines [3], but clusters identified during the study period are still important to understand. Understanding the pattern of liver injury in AHUO is crucial to properly identify cases of this unexplained phenomenon.

The emergence of AHUO clusters in 2022 occurred consecutively with a resurgence of SARS-CoV-2 [4]. SARS-CoV-2 has been found to cause liver function impairment, especially in the context of “multi-system inflammatory syndrome in children” (MIS-C) and can severely impact the liver. This has led some to hypothesize that COVID-19 could be a cause of hepatitis in children [5] or the result of multifactorial insults, including SARS-CoV-2 [4]. Thus, it is important to determine if AHUO, as an entity with no clear cause, has a unique biochemical profile for COVID-19-related liver injury.

The pattern of liver chemistry changes could hold clues to differentiating SARS-CoV-2 and AHUO. Reports of the impact on the liver from both AHUO and SARS-CoV-2 are mostly limited to small case series and case reports. Thus, it is important that literature reporting liver chemistry patterns for AHUO and COVID-19 be systematically evaluated so patterns can be characterized and compared. Differentiating patterns of hepatic injury between these conditions could also help clinicians differentiate the two conditions that can cause major hepatic injury.

This study aims to identify the patterns in liver-relevant biochemical data from COVID-19 patients and AHUO. Additionally, this study aims to compare the patterns of relevant biochemical data between COVID-19 and AHUO.

## Review

Systematic review

**Figure 1:**
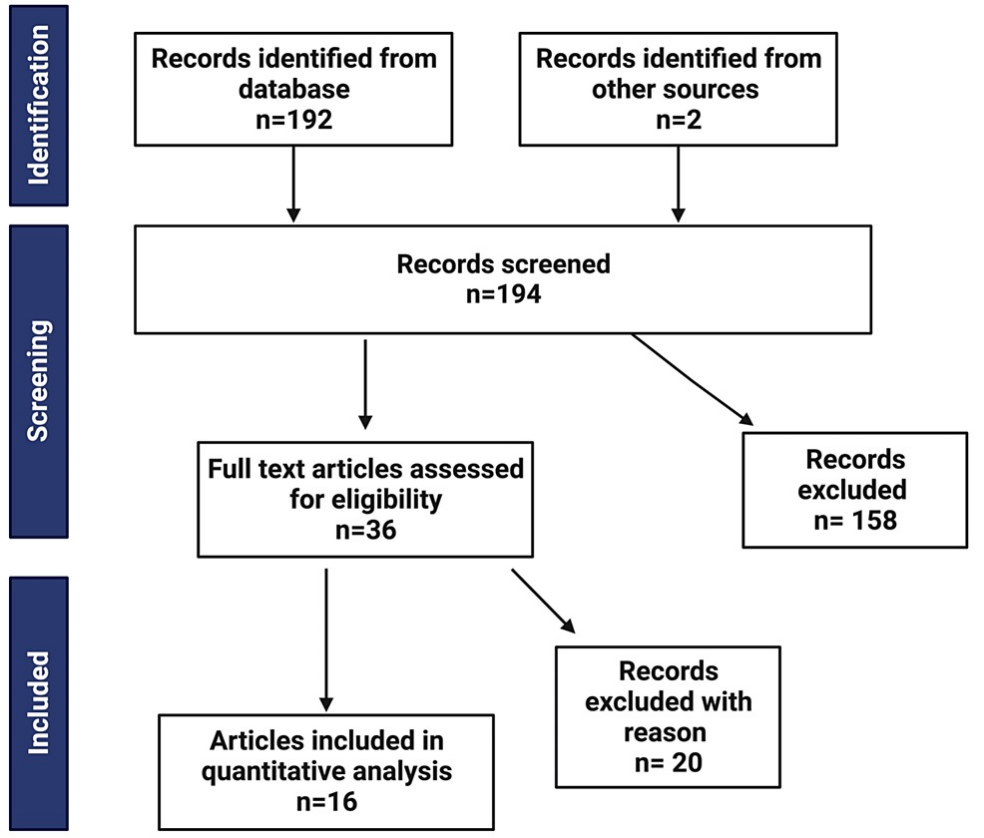
Flow chart representing the process for selecting articles for inclusion

Studies were identified by systematic review by search of MEDLINE (PubMed) using search terms “acute hepatitis of unknown origin children OR pediatric” and “covid-19 liver enzymes children OR pediatric.” Inclusion criteria were (1) pediatric patients (<=16 years old) with a diagnosis of AHUO and/or COVID-19 diagnosis by polymerase chain reaction, (2) case series, case reports, meta-analyses, and retrospective cohort studies with liver chemistries reported, (3) studies in English, and (4) published during or after 2020. Exclusion criteria were (1) patients >16 years old (as per the WHO Working Case Definition of AHUO), (2) a diagnosis of hepatitis A-E or auto-immune hepatitis and/or COVID-19 via serology only, and (3) no liver chemistries reported. Sixteen articles fit the inclusion criteria (Figure 1). Study sample sizes ranged from 1 to 294. Data was collected from studies published from March 2020 until December 2022. These studies are detailed in Tables 1, 2. These articles were grouped by AHUO patients and COVID-19 patients. Within the AHUO group, all patients were negative for COVID-19, except for 11/44 from the Kelgeri data set (see Table 1). The excluded articles are found in Table 3.

**Table 1:** Clinical and laboratory characteristics of acute hepatitis of unknown origin patients, normalized to multiples of the upper limit of normal *: Median (range), **: Mean (standard deviation) ALT: Alanine aminotransferase; AST: Aspartate aminotransferase; INR: International normalized ratio

Study	Cases	Sex	Age	ALT	AST	Total bilirubin	INR
Gutierrez Sanchez et al.* [6]	9	-	2.9 (6)	68.84 (163.76)	44.6 (80.75)	1.93 (1.9)	1.1 (5.73)
de Kleine et al.** [7]	26	-	5.8 (3.96)	109 (84.85)	-	12.6 (66.86)	2.08 (5.22)
Kelgeri et al.* [8]	44	-	4 (6)	114.32 (227.04)	-	1.17 (2.4)	-
Leiskau et al.** [9]	28	-	6.44 (5.03)	140.63 (110)	62.86 (59.99)	1.55 (1.48)	2.82 (1.7)
Zhou et al. [10]	1	M	10.5	93.2	36.8	8.24	-
Cardoso et al. [11]	1	F	1.8	32.56	26.43	-	-
Deep et al. [2]	1	-	2.6	180.72	128.2	35.57	3.09
	1	-	5	144.16	95.45	49.57	4.54
	1	-	5.1	151.56	88.77	55.57	4.44
	1	-	3.11	155.08	109.93	58.57	8.96
	1	-	2.5	194.16	111.27	42	7.09
	1	-	2.9	100.84	78.29	33.57	3.65
	1	-	2.11	150.8	149.7	52.57	5.36
	1	-	1.8	133.8	76.02	29.57	3.36
Lexmond et al. [12]	1	-	3	160	-	23.57	6.09
	1	M	8	88	-	30.57	1.82
	1	F	1.5	100	56.82	22.28	6.09
	1	F	2	144	-	11.85	3.63
Morita et al. [13]	1	M	10	71.36	39.69	2.07	1

**Table 2:** Clinical and laboratory characteristics of COVID-19 patients, normalized to multiples of the upper limit of normal *: Median (IQR); **: Mean (standard deviation) ALT: Alanine aminotransferase; AST: Aspartate aminotransferase; INR: International normalized ratio

Study	Cases	Sex	Age	ALT	AST	Total bilirubin	INR
Feng et al. [14]	1	F	0.25	1.00	1.00	-	-
	1	M	1.00	1.00	1.00	-	-
	1	M	7.00	0.68	0.92	-	-
Antala et al. [15]	1	F	0.50	193.69	64.28	1.02	6.73
	1	M	0.30	275.15	150.90	-	4.54
	1	F	16.00	506.80	239.40	-	2.27
	1	M	11.00	77.28	23.60	-	1.09
Zhou et al. [16]	1	M	17.00	0.71	0.94	-	-
	1	F	10.90	0.31	0.59	-	-
	1	F	10.90	0.79	0.76	-	-
	1	M	9.00	1.05	0.55	-	-
	1	F	0.58	3.03	2.12	-	-
	1	F	6.00	0.54	0.56	-	-
	1	F	0.40	1.21	0.76	-	-
	1	F	4.00	0.76	0.64	-	-
	1	M	8.00	0.72	0.56	-	-
	1	M	5.00	0.56	0.77	-	-
	1	F	3.00	0.24	1.02	-	-
	1	F	7.00	0.56	0.83	-	-
	1	F	3.00	0.44	0.95	-	-
	1	M	1.00	0.70	1.45	-	-
	1	F	3.00	1.72	0.82	-	-
	1	M	4.00	0.60	0.84	-	-
	1	F	0.25	2.55	1.49	-	-
	1	M	1.08	1.00	3.25	-	-
	1	M	0.08	1.00	1.00	-	-
	1	M	10.00	0.96	0.78	-	-
	1	F	0.25	1.05	0.89	-	-
	1	M	11.00	0.52	0.67	-	-
	1	M	10.00	0.88	0.60	-	-
	1	M	13.00	0.67	0.60	-	-
	1	F	5.00	0.48	0.73	-	-
	1	M	16.00	0.45	0.43	-	-
	1	F	14.00	0.64	0.62	-	-
	1	M	10.00	3.08	0.97	-	-
Chen et al. [17]	1	M	1.83	36.59	10.53	0.80	-
Alkan et al.* [18]	247	-	10.41 (3.79-15.38)	0.6 (0.48-0.80)	0.78 (0.64-1.07)		1 (0.1)**
	47	-	7 (1.38-14.75)	0.67 (0.5-0.85)	0.91 (0.72-1.16)		1 (0.1)**
Chao et al.* [19]	33	-	3.6 (0.1-17.2)	2.15 (1-5.52)	2.08 (1-3.11)		
Lexmond et al. [12]	1	-	3	160	-	23.57	6.09

**Table 3:** Articles excluded with reason AHUO: Acute hepatitis of unknown origin; PCR: Polymerase chain reaction

Title	Author/year/reference	DOI	Reason for exclusion
COVID-19-induced hepatic injury: a systematic review and meta-analysis	Abdulla et al., 2022 [20]	10.7759/cureus.10923	Patients >16
COVID-19 and the liver: overview	Amin, 2022 [21]	10.1097/MEG.0000000000001808	Patients >16
Abnormal liver function tests in patients with COVID-19: relevance and potential pathogenesis	Bertolini et al., 2020 [22]	10.1002/hep.31480	No reported liver chemistries
COVID-19: abnormal liver function tests	Cai et al., 2020 [23]	10.1016/j.jhep.2020.04.006	Liver chemistries not reported in a usable way
Long COVID-19 liver manifestation in children	Cooper et al., 2022 [24]	10.1097/mpg.0000000000003521	No diagnosis of COVID-19 by PCR
Liver chemistries in severe or non-severe cases of COVID-19: a systematic review and meta-analysis	Dong et al., 2022 [25]	10.4254/wjh.v14.i12.2012	Age of patients unclear
Risk factors for severe and critically ill COVID-19 patients: a review	Gao et al., 2021 [26]	10.1111/all.14657	Patients >16
Liver dysfunction and SARS-CoV-2 infection	Gracia-Ramos et al., 2021 [27]	10.3748/wjg.v27.i26.3951	Unclear patient age, Liver chemistries not reported in a usable way
Abnormal liver tests in COVID-19: a retrospective observational cohort study of 1,827 patients in a major U.S. hospital network	Hundt et al., 2020 [28]	10.1002/hep.31487	Patients >16
COVID-19-associated liver injury: clinical characteristics, pathophysiological mechanisms and treatment management	Li et al., 2022 [29]	10.1016/j.biopha.2022.113568	No liver chemistries reported
Liver injury is associated with severe coronavirus disease 2019 (COVID-19) infection: a systematic review and meta-analysis of retrospective studies	Parohan et al., 2020 [30]	10.1111/hepr.13510	Patients >16
Characteristics and outcomes of acute hepatitis of unknown etiology in Egypt: first report of adult adenovirus-associated hepatitis	Ramadan et al., 2022 [31]	10.1007/s15010-022-01945-1	Patients >16
Clinical characteristics of imported cases of coronavirus disease 2019 (COVID-19) in Jiangsu province: a multicenter descriptive study	Wu et al., 2020 [32]	10.1093/cid/ciaa199	Patients >16
Severe acute hepatitis of unknown aetiology in children - what is known?	Khader et al., 2022 [33]	10.1186/s12916-022-02471-5	No liver chemistries reported
Pediatric acute severe hepatitis of unknown origin: what is new?	Li et al., 2022 [34]	10.14218/JCTH.2022.00247	Duplicate cases
Investigation into cases of hepatitis of unknown aetiology among young children, Scotland, 1 January 2022 to 12 April 2022	Marsh et al., 2022 [35]	10.2807/1560-7917.Es.2022.27.15.2200318	Liver chemistries not reported
Acute hepatitis and adenovirus infection among children - Alabama, October 2021-February 2022	Baker et al., 2022 [36]	10.15585/mmwr.mm7118e1	Duplicate cases
Potential effects of SARS-CoV-2 on the gastrointestinal tract and liver	Lei et al., 2021 [37]	10.1016/j.biopha.2020.111064	Patients >16
COVID-19 and pediatric fatty liver disease: Is there interplay?	Di Sessa et al., 2021 [38]	10.3748/wjg.v27.i22.3064	No AHUO diagnosis

Data extraction

Screening of articles was completed by two independent reviewers with group discussion resolving any disparity in the collection. Data was collected from eligible sources from the text, figures, tables, and supplementary material by an independent reviewer. For eligible articles, the following items were recorded: author, sample size, age, sex, and liver chemistry-related values: alanine aminotransferase (ALT), aspartate aminotransferase (AST), total bilirubin, direct bilirubin, international normalized ratio (INR) of prothrombin time levels, and diagnosis of AHUO and/or COVID-19. All data generated during this study are included in this published article and its supplementary information files. Data extracted from the included studies is also available in the supplementary information files.

Statistical analysis

Statistical analysis was performed using custom R programming language (version 4.1.2, R Foundation for Statistical Computing, Vienna, Austria) scripts. Thresholds for the upper limit of normal (ULN) were dependent on age using values set by the Caliper project [39]. Liver enzymes were then normalized to multiples of the ULN. Raw clinical data were presented as mean and standard deviation, median and range, or median and interquartile range (IQR). For studies that reported summary data for multiple cases, summary data was used to expand summary data into a normalized, parametric data set using the r.norm function. This allowed for a combination of this data with other study data to allow for further summary statistics. The expanded data is used only for summary statistics and not presented herein as individual data points. Laboratory data was summarized as the median and IQR of the complete data set for both the COVID-19 group and the AHUO group. A comparison of means was performed using an unpaired two-sample t-test. Univariate linear models were used to correlate clinical characteristics with liver bloodwork parameters. Differences were considered significant when the p-value was <0.05.

Laboratory characteristics of acute hepatitis of unknown origin

Within the sample of AHUO (n=122), the median age was 3.93 years (IQR, 1.90-4.58). All patients included in this group tested negative for current SARS-CoV-2 infection, except for 11/44 patients from the Kelgeri et al. dataset (see Table 3). Median serum levels of ALT (n=96) were 116.33 (IQR, 32.56-181.08) multiples of the ULN. Median serum levels of AST (n=75) were 75.85 (IQR, 25.85-120.70) multiples of the ULN. INR levels (n=48) were also found to be consistently elevated, with a median INR of 2.962 (IQR, 1.81-5.36) multiples of the ULN. Median total bilirubin (n=121) was 1.87 (IQR, 0.12-4.03) multiples of the ULN.

Laboratory characteristics of COVID-19

For COVID-19 patients (n=380), the median age was 10.02 years (IQR, 3.45-9.66). Median serum levels of ALT (n=378) were 0.64 (IQR, 0.45-0.88) multiples of the ULN, and median serum AST levels were 0.83 (IQR, 0.63-1.05) multiples of the ULN. Median INR (n=299) serum level was 0.99 (IQR, 0.94-1.04) multiples of the ULN. The highest levels of AST, ALT, and INR were observed in a study by Antala et al. (see Table 3). This study followed four COVID-19-positive patients with acute hepatitis.

Comparison of acute hepatitis of unknown origin and COVID-19

**Figure 2:**
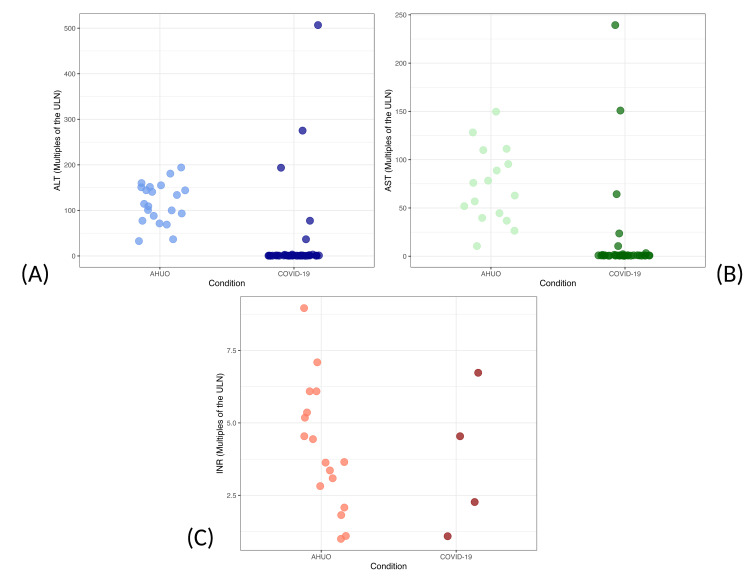
Acute hepatitis of unknown origin (AHUO) and COVID-19 liver chemistry changes occur in distinct patterns (A) Scatterplot of alanine aminotransferase (ALT) values illustrating that AHUO cases (n=21) had statistically higher values than COVID-19 cases (n=36). AHUO cases showed a smaller range than the COVID-19 cases. (B) Scatterplot of aspartate aminotransferase (AST) values demonstrating that AHUO cases (n=17) had statistically higher values than COVID-19 cases (n=36). (C) Scatterplot of international normalized ratio (INR) values demonstrating that AHUO cases (n=17) had statistically higher values than COVID-19 cases (n=4).

All patients in the pediatric AHUO group had elevated levels of ALT, AST, and INR. The mean difference between AHUO and COVID-19 patient ALT serum levels was 118.45 multiples of the ULN (p-value<0.05, 95% CI: 92.16 to 144.75) (Figure 2A). The mean difference between AHUO and COVID-19 patient AST serum levels was 78.51 multiples of the ULN (p-value<0.05, 95% CI: 59.988 to 97.02) (Figure 2B). The mean difference in INR levels between AHUO and COVID-19 patients was 2.52 multiples of the ULN (p-value<0.05, 95% CI: 1.83 to 3.211) (Figure 2C). It should also be noted that while the mean liver chemistries for these three tests were higher in the AHUO group, the range in the COVID-19 group was wider for ALT and AST, with a small number of COVID-19 patients having higher levels of these enzymes.

**Figure 3:**
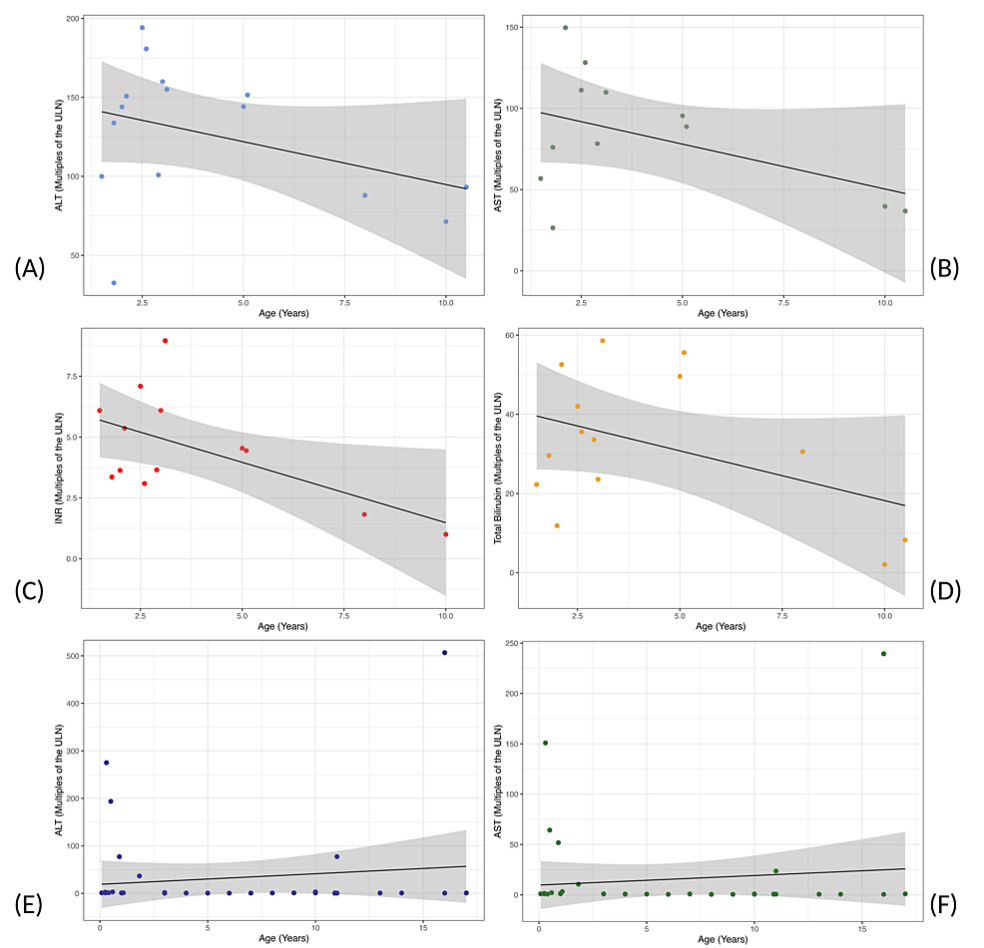
Liver chemistry changes are not different across the age range for patients with acute hepatitis of unknown origin (AHUO) and COVID-19 Scatterplots demonstrating that for AHUO patients there is no significant correlation between age and (A) alanine aminotransferase (ALT, n=17), (B) aspartate aminotransferase (AST, n=14), (C) international normalized ratio (INR, n=14), or (D) total bilirubin (n=16). Scatterplots demonstrating that, similarly, there is no significant correlation between age and (E) ALT (n=36) and (F) AST (n=36) in patients with COVID-19. Each line represents a linear regression, with the shaded area representing the 95% confidence interval. All liver chemistry values are normalized to multiples of the upper limit of normal (ULN).

Univariate linear correlation models were created stepwise for a variety of reported clinical characteristics of AHUO or COVID-19 and age, as shown in Figure 3. The correlation coefficient (R) values for the AHUO group were as follows: ALT (0.021; 95% CI: -0.18 to 0.21) (Figure 3A), AST (-0.029; 95% CI: -0.25 to 0.20) (Figure 3B), INR (0.040; 95% CI: -0.22 to 0.23) (Figure 3C), and total bilirubin (-0.050; 95% CI=-0.22 to 0.12) (Figure 3D). R values for the COVID-19 group were as follows: ALT (-0.014; 95% CI=-0.12 to 0.087) (Figure 3E) and AST (-0.018; 96% CI=-0.12 to 0.08) (Figure 3F). There were no statistically significant correlations between age and any of the analyzed parameters for either condition.

Discussion

This study found, through a systematic review of more than a dozen studies, that the pattern of COVID-19 and AHUO changes to liver chemistries are distinct. AHUO produced substantially more elevated levels of liver chemistries such as AST, ALT, INR, and total bilirubin. However, there was a subset of COVID-19 cases where liver chemistries were highly elevated. There was also no significant correlation with age (across the pediatric age range) for individual liver chemistry tests for either condition. In summary, these findings indicate that the impact of COVID-19 on the liver occurs in a pattern different than that of AHUO. This suggests that active COVID-19 infection alone does not directly cause AHUO; however, COVID-19 may still be a contributory factor [40]. This finding is in keeping with the previous findings of Servellita et al., Morfopoulou et al., and Ho et al., which provide potential evidence that adeno-associated virus 2 (AAV2), rather than COVID-19 may be linked to the AHUO outbreak [41-43]. Further investigation into this topic is required to provide conclusive evidence that COVID-19 is not a contributory factor in AHUO [40].

Cardoso et al. and Morita et al. show the pattern of biochemical changes in patients with AHUO has a predominantly mixed hepatocellular and cholestatic pattern of liver injury, with hepatocellular patterns being more widely documented [11,13]. However, these studies understandably suffer from the limitation of small sample sizes, thus we used multiple published sources to increase the sample size to re-examine the data. Similarly, with COVID-19-associated liver disease, Antala et al. and Lazova et al. showed a mixed hepatocellular and cholestatic pattern of substantial liver injury [15,44]. This presentation of acute COVID-19 is similar to the known pattern of liver injury in the rare COVID-19-associated disease MIS-C, although MIS-C liver injury is by nature multisystemic as well [44].

Similar to our findings, AHUO has previously been shown to impact both INR and bilirubin in a variable manner, with a wide range of effects [2,12]. Many previous reports of AHUO cases showed a considerable elevation in these indicators [2,12]. Similarly, COVID-19 cases with liver injury also feature elevation in INR, total bilirubin, and lowered albumin, although with a lower range of elevation [15,44]. Thus, there does not appear to be a specific pattern of liver biochemical marker changes that could reliably differentiate AHUO and COVID-19-induced liver injury. This finding is not unexpected, as few liver diseases can be differentiated by their impact on liver chemistry alone.

Despite the substantial overlap in the patterns of AHUO and COVID-19-induced liver injury, certain patterns suggested by the present study data are worth highlighting. AHUO patients' elevations in ALT and AST are relatively consistently elevated in the range of 0-200 multiples of the ULN, centralized around 75-100. In contrast, COVID-19 patient elevations in these enzymes appear more diffusely spread over a wider range. Additionally, there is minimal separation between the changes to liver function tests (INR, bilirubin, and albumin) between the two conditions. Thus, if further data were to support these trends, it may be found that if patients had ALT and AST elevations outside of this apparent AHUO elevation range, that would more strongly suggest a non-AHUO cause of liver injury.

It is also important to note that the elevations in ALT were relatively higher than AST in both AHUO and COVID-19 patients. Determination of whether ALT or AST is more elevated is a step often used by clinicians to narrow down potential causes of liver injury in an individual patient. Classically, ALT is thought to be more highly elevated in virus-driven injury. Since ALT was more elevated than AST, this supports the possible etiology of AHUO as a virus-related phenomenon.

In March 2023, three studies potentially linked the AHUO global outbreak to AAV2 [41-43]. In these studies, most affected participants had detectable levels of AAV2 in blood and liver samples, compared to low levels in control participants [3]. Despite the possible linkage to AAV2, no study has yet been able to definitively rule out COVID-19 as a contributory factor to AHUO, due to the coincided timing of the outbreaks and the high prevalence of SARS-CoV-2 in the population at this time [3]. Further research should focus on SARS-CoV-2 proteins as possible superantigens that can trigger a potent immune response to AAV2, subsequently leading to AHUO.

Some limitations are present in this study. First, selection bias is an important consideration as our study is more likely biased toward cases that occurred early in the identification period for both AHUO and COVID-19. This is because the novelty of both conditions likely led initially to more publications. Then, over time, it is less likely that a pediatric patient with liver chemistry alterations would be reported in the literature. There is also potential for selection bias for more severe liver chemistry changes in this study, since milder liver chemistry changes are less likely to be noteworthy, and thus less likely to be reported in the literature. Second, the data set was predominantly comprised of cases <7.5 years old, although the WHO working case definition allowed cases up to 16 years old. Thus, the findings may not be fully applicable to older pediatric patients.

## Conclusions

In conclusion, by combining data from multiple studies, it appears that the pattern of liver injury due to AHUO is a relatively consistent range of ALT-predominant hepatocellular patterns. Liver injury due to COVID-19 was uncommon in the study population but the range of liver enzyme elevation was higher than the AHUO values, although still in an ALT-predominant hepatocellular pattern. It is worth noting that multiple patients in the study population had similar patterns of liver enzyme elevation in the same relative range, despite having different diagnoses. Thus, differentiation of AHUO and COVID in patients cannot be accomplished based solely on the liver laboratory parameters discussed, an important consideration for clinicians encountering a patient with liver chemistry values in this range. Further research into the highly elevated liver chemistries seen in a small population of COVID-19 patients is needed.
